# Effects of Temperature and Wildflower Strips on Survival and Macronutrient Stores of the Alfalfa Leafcutting Bee (Hymenoptera: Megachilidae) Under Extended Cold Storage

**DOI:** 10.1093/ee/nvac062

**Published:** 2022-08-14

**Authors:** Mia G Park, Casey M Delphia, Cassandra Prince, George D Yocum, Joseph P Rinehart, Kevin M O’Neill, Laura A Burkle, Julia H Bowsher, Kendra J Greenlee

**Affiliations:** Department of Biological Sciences, North Dakota State University, Fargo, ND, USA; Department of Land Resources and Environmental Sciences, Montana State University, Bozeman, MT, USA; Montana Entomology Collection, Marsh Labs, Montana State University, Bozeman, MT, USA; Edward T. Schafer Agricultural Research Center, Biosciences Research Laboratory, Fargo, ND, USA; Edward T. Schafer Agricultural Research Center, Biosciences Research Laboratory, Fargo, ND, USA; Edward T. Schafer Agricultural Research Center, Biosciences Research Laboratory, Fargo, ND, USA; Department of Land Resources and Environmental Sciences, Montana State University, Bozeman, MT, USA; Department of Ecology, Montana State University, Bozeman, MT, USA; Department of Biological Sciences, North Dakota State University, Fargo, ND, USA; Department of Biological Sciences, North Dakota State University, Fargo, ND, USA

**Keywords:** alfalfa leafcutting bee, fluctuating thermal regime, insect storage, macronutrients, wildflower strips

## Abstract

*Megachile rotundata* (F.) is an important pollinator of alfalfa in the United States. Enhancing landscapes with wildflowers is a primary strategy for conserving pollinators and may improve the sustainability of *M. rotundata*. Changing cold storage temperatures from a traditionally static thermal regime (STR) to a fluctuating thermal regime (FTR) improves overwintering success and extends *M. rotundata’s* shelf life and pollination window. Whether floral resources enhance overwintering survival and/or interact with a thermal regime are unknown. We tested the combined effects of enhancing alfalfa fields with wildflowers and thermal regime on survival and macronutrient stores under extended cold storage (i.e., beyond one season). *Megachile rotundata* adults were released in alfalfa plots with and without wildflower strips. Completed nests were harvested in September and stored in STR. After a year, cells were randomly assigned to remain in STR for 6 months or in FTR for a year of extended cold storage; emergence rates were observed monthly. Macronutrient levels of emerged females were assessed. FTR improved *M. rotundata* survival but there was no measurable effect of wildflower strips on overwintering success or nutrient stores. Timing of nest establishment emerged as a key factor: offspring produced late in the season had lower winter survival and dry body mass. Sugars and glycogen stores increased under FTR but not STR. Trehalose levels were similar across treatments. Total lipid stores depleted faster under FTR. While wildflowers did not improve *M. rotundata* survival, our findings provide mechanistic insight into benefits and potential costs of FTR for this important pollinator.

Inadequate food resources associated with intensified agriculture and development are a major factor contributing to pollinator declines (Potts *et al.* 2010; Goulson *et al.* 2015). Bees require carbohydrate-rich floral nectar to fuel adult flight and protein- and lipid-containing pollen to support offspring growth and development (Herbert 1992). While food quantity has clear effects on bee survival, development, and reproduction (Haydak 1970; Keller *et al.* 2005; Brodschneider and Crailsheim 2010), more recent work reveals the importance of resource quality on bee health. Diverse, multi-species pollen diets increase social immunity for honey bees compared to single-species pollen diets, even when protein content is kept constant (Alaux *et al.* 2010). Floral diversity increases opportunities for bees to take advantage of flowers with pharmacological properties, as when bumble bees self-medicate on sunflower pollen to reduce gut parasite loads (Richardson *et al.* 2015). By comparing colonies near habitats with or without floral resource enhancements, field studies corroborate benefits of abundant and/or diverse forage on honey bee and bumble bee health and, ultimately, colony growth and overwintering survival (Westphal *et al.* 2009; Alaux *et al.* 2017; Schulte *et al.* 2017; Ricigliano *et al.* 2019).

Our understanding of nutrition effects on bee fitness and health is largely based on investigations of social honey bees and bumble bees (Family Apidae). Most (~90%) bee species, however, are not social but solitary (O’Toole and Raw 1991), meaning each female creates and provisions her own nest for a few weeks to months with no assistance from others. A female lays an egg in a cell she has provisioned with a ball of pollen and nectar and then closes it off; the egg hatches and the larva develops on the cell provision. Larval size and energy stores depend directly on the size and nutritional composition of the provision. A solitary female bee is the sole forager for her offspring and typically has a smaller foraging range (Gathmann and Tscharntke 2002). Consequently, offspring success of solitary bees may be more sensitive to temporal and spatial patchiness and/or composition of floral resources than social species, for which multiple adult workers can collect pollen from a diversity of resources. Despite a known physiological link between nutrition and bee fitness, surprisingly few experiments have focused on non-Apidae pollinators.

Larval nutrition is particularly important for temperate bees, which need adequate energy reserves to successfully complete a long overwintering period. Most solitary bees spend much of their life cycles overwintering in dormant states of diapause until they emerge as adults the following year. Diapausing insects employ several adaptations to tolerate cold temperatures; however, winter chill injury and death still occur (Hahn and Denlinger 2007; Colinet *et al.* 2018). While abiotic stresses are primary drivers of overwintering mortality, nutrition also plays a key role (Hahn and Denlinger 2007, 2011; Sinclair 2015). Insects depend on macronutrients for energy and physiological function. Lipids and glycogen are notably important as energy stores during overwintering: they increase in individuals readying for diapause and are linked to overwintering success (Hahn and Denlinger 2007). Resource depletion affects overwintering individuals directly through starvation or indirectly by reducing reserves needed to fuel physiological processes leading to stress tolerance (Hahn and Denlinger 2007) and availability of cryoprotectants (Sinclair 2015). Overwintering success has important implications for wild populations in nature, but also for managed pollinators for whom diapause occurs under artificial conditions. Artificial cold storage is often unnaturally static and, likely, stressful (Colinet *et al.* 2018).

Introducing fluctuating temperatures in artificial cold storage increases overwintering success of various insects (reviewed in Colinet *et al.* 2018). Adding a daily warm pulse, through a fluctuating thermal regime (FTR), allows organisms to resume physiological processes that prevent or repair chill injury. However, FTR may incur fitness costs if it depletes energy reserves faster than a static thermal regime (STR). Compared to STR, FTR has been associated with lower lipid content, body mass, fecundity, and longevity of some insects (Carroll and Quiring 1993; Ismail *et al.* 2010; Marshall and Sinclair 2010; Basson *et al.* 2012). For other insects, no differences in fecundity (Murdoch *et al.* 2013) or flight performance and metabolism (Bennett *et al.* 2013) have been observed. Comparative studies that track macronutrient stores through overwintering may help us better understand the net benefits and costs of FTR and the role nutrition plays in overwintering success.

Among pollinators, the solitary alfalfa leafcutting bee, *Megachile rotundata* (F.), is a good model organism to study effects of FTR and energetics on overwintering success. *Megachile rotundata* nests can be purchased as loose leaf cells and placed in plots of varying floral resource levels, and adult bees generally do not fly more than ca. 300 m from nesting sites (Kesoju *et al.* 2021). Newly completed nests are easily monitored and transported back to the laboratory for cold storage. *Megachile rotundata* is managed primarily for alfalfa seed pollination and is of great economic importance worldwide (Pitts-Singer and Cane 2010). Unlike managed, social bees with foraging seasons that last half the year, *M. rotundata* is active for only 6–8 weeks during summer months. Therefore, artificially extending the overwintering period—or shelf-life—of *M. rotundata* is of great interest to alfalfa leafcutting bee managers as it would provide increased flexibility in the timing of adult release and the number of crops for which *M. rotundata* could be used for pollination services. Under traditional, static cold storage conditions, mortality is high when prepupae are overwintered beyond their typical winter cycle; however, FTR dramatically increases survival of *M. rotundata* under extended cold storage, without compromising adult performance, as measured by flight and longevity (Bennett *et al.* 2013; Rinehart *et al.* 2013). The fact that lipid stores accrued during larval development are clearly important later to adult female *M. rotundata*, as the reserves decline rapidly after adult emergence while females are developing eggs (O’Neill *et al.* 2015), also makes this an interesting model to study macronutrients stores under extended cold storage.

While the above-mentioned performance studies examined offspring pooled over an entire nesting season from the same environment, a more resolved understanding of how timing of *M. rotundata* nesting and floral resource availability influences FTR benefits is lacking. Because nesting activity of *M. rotundata* typically lasts up to 2 months, offspring provisioned earlier in the nesting season spend several weeks longer in a pre-dormant, prepupual stage compared to those provisioned later in the season. Consequently, energy stores may be more depleted among these early bees, leading to increased mortality during diapause. While previous work demonstrates the importance of environmental history for *M. rotundata* diapause success, little has been done to isolate specific environmental factors that are most important (Yocum *et al.* 2018). From a practical perspective, a better understanding of the effects of various environmental factors on diapause is needed as reproduction of *M. rotundata* in the United States is unsustainable, given a low replacement rate. Increased availability of late season flowers after a mass blooming crop, like alfalfa, can increase the abundance of trap-nesting bees (Dainese *et al.* 2018). Supplementing alfalfa fields with wildflower strips is, therefore, a possible measure to increase resource availability to *M. rotundata* and, in turn, overwintering success and fitness.

Here we couple field and laboratory experiments to investigate cold stress and nutrition dynamics during extended cold storage of *M. rotundata*. *Megachile rotundata* offspring from experimental alfalfa plots with and without wildflower strips were exposed to STR or FTR after one year in static cold storage, and resulting emergence successes and macronutrient levels were measured. We predicted that (1) supplemental wildflower strips will increase *M. rotundata* macronutrient stores and overwintering success; (2) nutrient stores will diverge between FTR and STR under extended cold storage with greater depletion of energy reserves under FTR; and (3) offspring produced late in the season will have higher energy stores and overwintering success.

## Methods

### Study Organism

Adult *Megachile rotundata* actively nests during mid to late summer. Offspring complete the larval stage after a few weeks and overwinter as prepupae until the next summer. In commercial operations, nests are typically harvested in September and kept in artificial cold storage at 4–6°C, for 7–9 months (Pitts-Singer and Cane 2010). Agricultural managers break diapause in the spring by moving bees to 29–30°C (Pitts-Singer 2008). The time to the emergence of females at those temperatures is typically 3 weeks (O’Neill *et al.* 2011).

### Field Design

**Figure 1.**
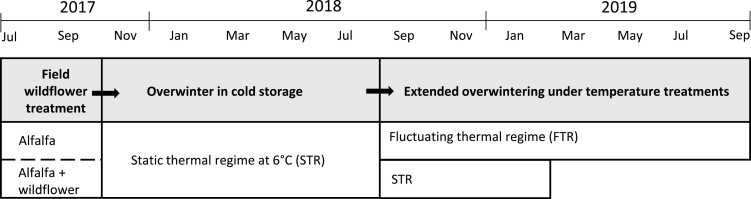
Study timeline and design. The field component of this study exposed adult *Megachile rotundata* to experimental alfalfa plots with or without adjacent wildflower strips. Offspring produced during the field study were harvested and brought to the lab where they overwintered in constant 6°C (STR), as is typical of commercial operations. After a year, quiescent prepupae were placed in extended cold storage under one of two temperature treatments: continued STR or fluctuating thermal regime (FTR). FTR is similar to STR except that a 20°C warm pulse is introduced daily for 1 h. Subsets of bees were incubated at 29°C monthly during temperature treatments, including baseline *t* = 0; emergence success, sex, and time to emergence were recorded. Bees were frozen within 24 h of emergence, freeze dried, and analyzed for macronutrient stores.

Please see Figure 1 for a schematic of treatments and the timing of the study. *Megachile rotundata* brood cells were collected from a field experiment conducted in 2017 at Lutz Farm (lat. 45.8132°N, long. 111.0550°W), a Montana State University research farm comprised of primarily wheat, barley, and other small grains, and located near Bozeman, MT, USA. Each of the six research plots was comprised of 0.05 hectares (23 m × 23 m) dryland alfalfa (variety Cooper) planted on three-foot centers for a total of 676 plants per plot. Next to half of the alfalfa plots, wildflower strips (4.5 m × 25 m) were established along one edge. Wildflower strips were comprised of species that would bloom after alfalfa’s peak bloom and do not have seeds that could be confused with that of alfalfa, which could decrease alfalfa seed purity if wildflowers became established in alfalfa fields (Table 1 for plant list). To ensure the independence of research plots, all plots were spaced at least 350 m apart (Van Deynze *et al.* 2008; Zurbuchen *et al.* 2010); mark recapture confirmed that *M. rotundata* did not move among plots.

**Table 1.** Plant species included in supplemental wildflower strips adjacent to alfalfa plots.

Wildflower species	Common name
*Calendula officinalis* L.	Common marigold
*Clarkia pulchella* Pursh	Deerhorn clarkia
*Coreopsis tinctoria* Nutt.	Plains coreopsis
*Cosmos bipinnatus* Cav.	Garden cosmos
*Helianthus annuus* L.	Common sunflower
*Heliomeris multiflora* Nutt.	Showy goldeneye
*Phacelia campanularia* A. Gray	Desertbells
*Phacelia tanacetifolia* Benth.	Lacy phacelia

*Megachile rotundata* from a 2016 commercial field population was purchased as loose cell bees from Seed Source Inc. (Toston, MT) and kept in the dark at 6°C from late winter until being reared for release beginning in June 2017. At that time, we moved the bees to room temperature for 24 h and then placed them in a 28°C growth chamber to break their diapause, so they would proceed in development from the pre-pupal to adult stage. On 03 July 2017, adult bees (ca. 6,000 cells) were released at each of six bee shelters placed at the center-edge of the six research plots. Nest holes for females were provided in sterilized wood laminate nesting blocks placed in wood shelters measuring 1.4 m tall × 1.2 m wide × 0.78 m deep (designed after O’Neill 2004); each shelter contained 40 nesting blocks that provided nearly 5,000 tunnels. Alfalfa floral density peaked in week 2 after bee release and steadily decreased during the study period. Wildflower strips began to bloom in week 5 and floral density increased with each week of the study period. Over the course of ca. 9 h of visual observations, female *M. rotundata* were recorded visiting planted wildflowers, though uncommonly (<20 individuals), and from only a few species.

The week of nest completion (based on the appearance of the final nest cap) was tracked by painting each capped nest with a color unique to each week. Nesting blocks were collected from the field in late September 2017, well after nesting activity had ceased; nests were removed from wood laminate blocks and stored as loose cells. Cells were placed in static cold storage (6°C) in late October of 2017 and maintained at this temperature until September 2018, at which time temperature treatments detailed below were initiated (Fig. 1).

### Temperature Treatments

In September 2018, after 11 months of cold storage at 6°C and darkness, brood cells produced in the field experiment were transferred to one of two thermal regimes: static thermal regime (STR) or fluctuating thermal regime (FTR, Fig. 1). As described in Rinehart *et al.* (2013), STR consisted of a 6°C constant temperature in darkness. FTR consisted of 21 h at 6°C, a 1 h ramp to 20°C (with a ramp speed of 0.23°C/min), 1 h incubation at 20°C, and a 1 h ramp down to 6°C, in darkness. Each temperature regime was conducted in a Percival model I-30BLL reach-in incubator. Up to 15 loose brood cells from each plot (*n* = 6, 3 plots per wildflower treatment) and week (*n* = 6) were individually placed into wells of 24-well culture plates. Brood cells were gently pressed between the thumb and forefinger to exclude soft cells that signaled non-viable pollen balls or dead larvae (Pitts-Singer and Cane 2010; O’Neill *et al.* 2011). All plates from a particular plot were organized on a single tray (hereafter referred to as a ‘plot-tray’). Exposure to thermal treatments continued to March 2019 for STR bees (3,240 total = 15 bees × 3 plots × 2 wildflower treatments × 6 weeks × 6 months) and to August 2019 for FTR bees (6,480 total = 15 bees × 3 plots × 2 wildflower treatments × 6 weeks × 12 months). At monthly intervals, six plot-trays from temperature regimes were incubated at 29°C in darkness, emulating standard protocol to initiate development leading to adult emergence (Pitts-Singer 2008). We began daily monitoring of emergence and sex 2.0–2.5 weeks after incubation (Pitts-Singer and Cane 2010). We dissected brood cells from which adults did not emerge to record the stage of death (i.e., prepupa, pupa, or adult).

### Bee Nutrition

To compare changing nutritional stores of bees exposed to temperature and wildflower treatments through extended cold storage, we quantified total lipids and several carbohydrates of newly-emerged adult female bees from temperature treatments. We analyzed the following carbohydrates: sugars (mono- and disaccharides), glycogen, and trehalose. Offspring produced early (2 weeks after initial release) and late (6 weeks after initial release) in the 2017 field season were frozen at −20°C within 24 h of adult emergence and freeze-dried prior to analysis. Early-season bees would have fed primarily on alfalfa pollen as larvae, regardless of the research plot. Late-season bees from research plots with wildflower strips would have had access to supplemental forage, while late-season bees from alfalfa-only research plots would have had access to alfalfa. All bees, regardless of plot and timing of the season, also had access to pollen from naturally occurring (or cultivated) wildflowers and weeds within the landscape, though these were relatively rare.

We adapted Giri *et al.* (2019) to extract carbohydrates and lipids from 225 *M. rotundata* females (7–10 bees × 2 temperature regimes × 2 wildflower treatments × 3 time periods × 2 weeks) with the following modifications: (1) freeze-dried bees were homogenized dry with zirconium beads in a Bullet Blender within a 2 mL polypropylene tube (Next Advance Inc., Troy, NY, USA); (2) homogenization continued for 5 min in 0.1 mL 2% sodium sulfate; (3) 1.120 mL 1:1 chloroform:methanol mixture was added to separate lipids from carbohydrates, (4) 0.4 mL deionized (DI) water was used to precipitate glycogen, (5) 0.8 mL DI water was added to separate lipids from sugars. We used the anthrone and vanillin-phosphoric protocols (Van Handel 1985a; b) to assay carbohydrates and lipids, respectively. We followed a recent modification of both protocols for microplates (M. Dillon, *pers. comm.*). For carbohydrates, we added a 5-min shaking step on a plate shaker (VWR International, Radnor, PA, USA) to ensure adequate mixing of the anthrone reagent before and after incubation. For lipids, we also added shaking steps after adding sulfuric acid and after adding the vanillin-phosphoric reagent, prior to reading the absorbency. Due to detection issues in microplates, we followed Van Handel (1985b) protocols to assay trehalose in glass culture tubes using anthrone. Macronutrient levels were standardized by freeze dried weight and presented as proportion dry weight (ug macronutrient/ug dry weight).

### Statistical Analyses

Statistical analyses were conducted with software R version 3.6.1 (R Development Core Team 2009). To test the effects of wildflower strips, time in cold storage, nest timing, and temperature regime on emergence success, we used generalized linear mixed-effects models (GLMMs) with binomial error distribution. While we followed the emergence of bees under FTR for a full year, through August 2019, we focused analyses on emergence data from September 2018 through March 2019, which marked the end of our STR experiment. We included a research *plot* as a random effect. Fixed effects included a number of months in overwinter storage (*month*), temperature regime (STR or FTR; *temperature*), presence/absence of wildflower strips (*wildflower*), and week during the field season when the nest was built (*week*). We included the following interactions of interest, defined a priori: *month* × *temperature*, *month* × *week*, *wildflower* × *week*, and *month* × *temperature* × *wildflower*. Based on work by Rinehart *et al.* (2013), we anticipated a strong *month* × *temperature* interaction, with a faster decrease in emergence success in STR. Because wildflower strips bloomed late in the season (weeks 5–7), we expected to see a significant *wildflower* × *week* interaction if wildflower strips affected emergence. A *month* × *week* interaction was included to explore Yocum *et al.*’s (2018) finding that gene expression and, therefore, physiology differs between offspring produced early and late in the season, and may lead to differential emergence through time. Finally, a three-way *month* × *temperature* × *wildflower* term was included to test whether effect of wildflower strips on overwintering success differs under STR and FTR. We used the *glmer* function in the *lme4* package (Bates *et al.* 2015) to run logistic regression models. We identified the best fit and most parsimonious model predicting emergence success, using small sample-corrected Akaike information criterion (Burnham and Anderson 2002). Given a strong divergence in emergence success between FTR and STR through time, all candidate models, except for the null model, included the main effects of *month* and *temperature*, as well as their interactions. To address heteroscedasticity, we included a quadratic and cubic term for months in storage within the emergence model. To address multicollinearity, we centered covariates on *month* and *week* in models. To see a summary of the top five selected models please see our supplementary data, Table S1.

We used parallel, beta regression mixed models (link = logit) to test the effects of wildflower strips, months in cold storage, week, and temperature regime on macronutrient levels in overwintering female bees. Beta regression is recommended for the analysis of continuous proportions bounded by 0 and 1 (Douma and Weedon 2019). We included the same predictor variables as described above in emergence models, except *months* in cold storage and *week* were treated as ordinal variables. We tested macronutrient levels for a subset of months at key moments of emergence success between STR and FTR: 12 months, September 2018, our baseline before FTR was applied; 14 months, November 2018, pre-divergence of emergence success; and 16 months, January 2019, post-divergence of emergence success. We focused on weeks 2 and 6 to represent early and late in the season, respectively. Field plot and microplate were included as random effects. We used the following variable dispersion factors to account for uneven residual variation among treatment levels: *month* × *week* × *temperature* × *wildflower* for sugars, trehalose, and total lipids models; *month* × *temperature* for glycogen model. As described above, AIC model selection was used to identify top models predicting macronutrient levels in female bees. We did not constrain models to include specific predictors. We used R packages *glmmTMB* (Brooks *et al.* 2017) to run beta regression models and *MuMin* (Bartoń 2022) to conduct AIC model selection. To validate all fitted models, we used the package *DHARMa* (Hartig 2021) to assess the uniformity and homoscedasticity of residuals. We note that our trehalose model is under dispersed. We retain this model because under dispersion results in overly conservative *P*-values, and this model distribution performed better than others we explored (i.e., negative binomial and gamma). We verified that collinearity among covariates was not an issue using the variance inflation factor (VIF ≤ 5.0; Zuur *et al.* 2010). To see a summary of the top five selected models, please see our supplementary data, Supplementary material Table S2–S5.

Finally, in R package *lme4* (Bates *et al.* 2015), we used a general linear mixed model, with the *plot* as a random variable, to test the influences of the following predictors on freeze-dried mass of female *M. rotundata* (*n* = 219) used in macronutrient testing: *month*, *temperature*, *wildflower*, *week*, *month* × *temperature*, *wildflower* × *week*. Tukeys adjusted post hoc, pairwise comparisons were conducted using package *lsmeans* (Lenth 2016).

We plot observed data with standard error bars and marginal effects, or predicted means, with 95% CI error bars.

## Results

### Overwintering success

**Figure 2.**
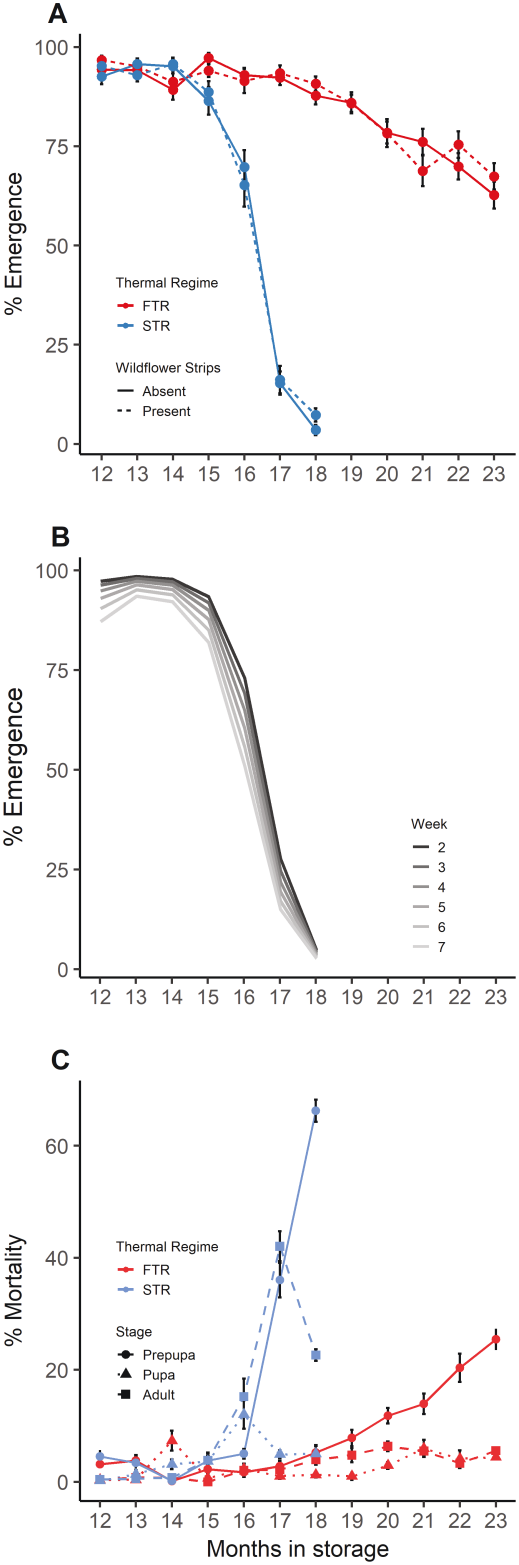
Effects of temperature regime, wildflower strips, and nest timing (week) on overwintering success of *Megachile rotundata* as months in cold-storage increased. (A) Observed emergence rates of bees nesting in alfalfa plots with (dashed lines) or without (solid lines) adjacent wildflower strips, under a static thermal regime (STR; blue) or fluctuating thermal regime (FTR; red). (B) The marginal effect of the week showed decreased overwintering survival the later in the season the offspring were produced (confidence intervals excluded to allow for clearer visualization). (C) Stage of development (prepupa, pupa, or adult) for those offspring that failed to emerge under STR (blue) or FTR (red). Data in figures A and C are means ± 1SE.

Temperature regime and nesting week influenced emergence success of *M. rotundata* under extended storage, but the presence/absence of wildflower strips did not. *Month* (linear, quadratic, and cubic), *temperature*, *week*, and *month* × *temperature* were included in our best model and were, therefore, the primary correlates of cold-storage survival (Supplementary Table S1). Offspring stored in FTR had increasingly higher probability of adult emergence with months in storage than those stored under STR (Table 2, *month* × *temperature*; Fig. 2a). Offspring provisioned earlier in the flight season were more likely to successfully emerge as adults than those produced later in the season (Table 2, *week*, Fig. 2b). *Wildflower* did not make it into the best fit model, indicating that supplementing alfalfa fields with wildflower strips had no measurable effect on *M. rotundata* emergence (Table 2, Supplementary Table S1). Total mortality was lower under FTR and occurred later in the storage period. Under STR, overwintering mortality of *M. rotundata* occurred at the prepupal or adult stage, whereas bees stored under FTR died largely as prepupae (Fig. 2c).

**Table 2.** Summary of the best fit mixed logistic regression model (link = logit) testing effects of months in storage (linear, quadratic, and cubic month), temperature regime (temp), and their interaction, as well as nest timing (week; offspring produced in week 2 or 6 of field season) on adult emergence success in overwintering *Megachile rotundata*. A random field plot term was included. Month and week were mean centered. STR is the reference temperature regime. Week 2 is the reference week.

Effect	Estimate	S.E.	*Z* value	*P* value
Month	−177.222	6.117	−28.97	**<**0.00001
Month^2^	−73.798	5.605	−13.17	**<**0.00001
Month^3^	17.124	5.772	2.97	0.00301
Temp	1.795	0.104	17.23	**<**0.00001
Week	−0.228	0.029	−7.86	**<**0.00001
Month × temp	155.471	8.650	17.97	**<**0.00001
Month^2 ^× temp	72.232	8.349	8.65	**<**0.00001
Month^3 ^× temp	−35.433	8.400	-4.22	0.00003
Month × week	0.0359	0.0157	2.29	0.02189

### Bee Nutrition

**Figure 3.**
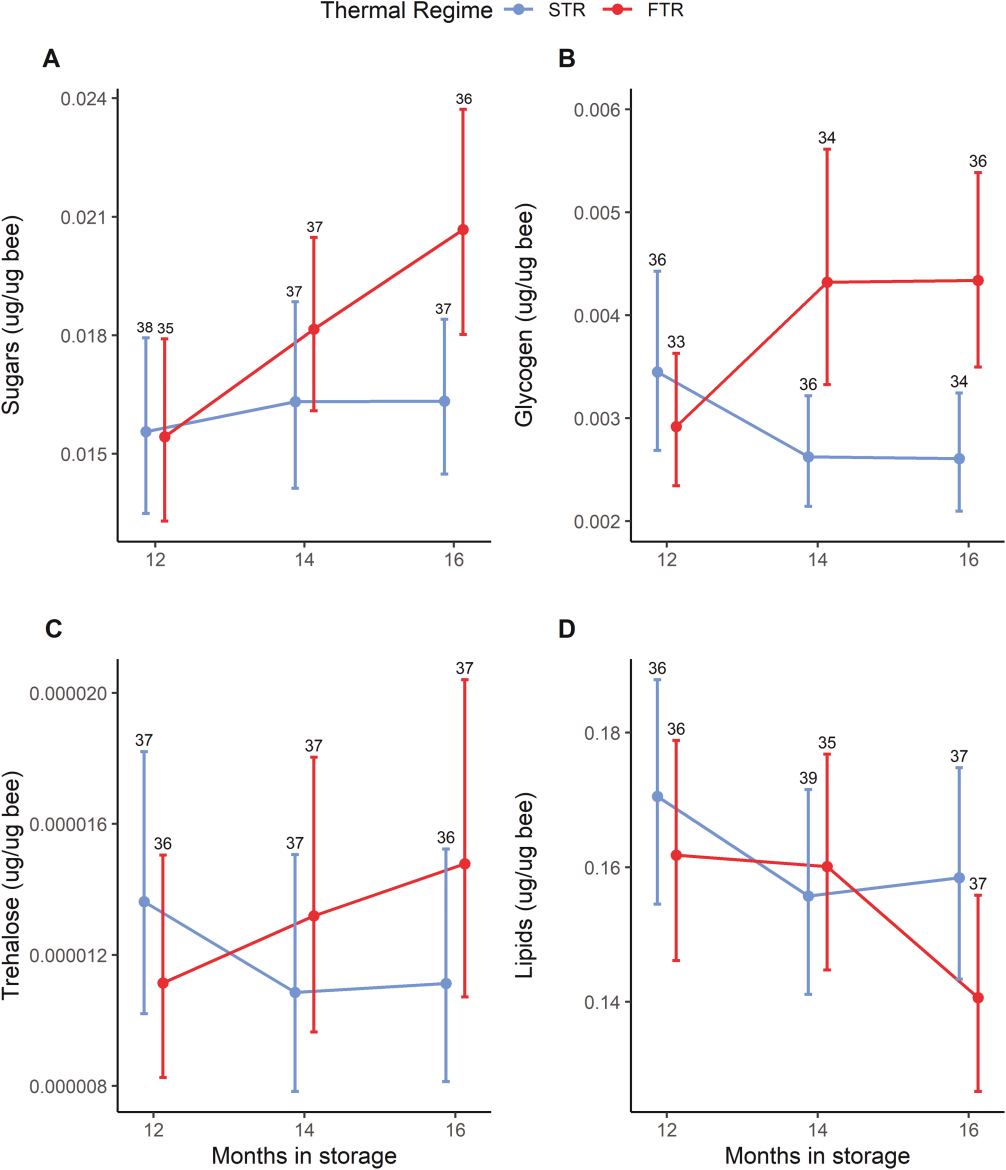
Marginal effects from mixed beta regression showing proportions (A) sugars, (B) glycogen, (C) trehalose, and (D) total lipids of newly emerged adult female *Megachile rotundata* from extended cold storage under two experimental thermal regimes: static (STR, blue) and fluctuating (FTR, red). (A) Proportion sugars include trehalose. Months in storage refers to time spent in cold storage; 12 months marks the beginning of temperature treatments. Week and forage treatments were fixed at 2 weeks and the absence of wildflower strips, respectively. Data are estimated means ± 95% CI. The numbers above the error bars represent sample sizes.

Except for trehalose, changes in macronutrient content were observed through time and were strongly associated with temperature regime (Fig. 3, Supplementary Table S2–S5). Proportion of sugars (Table 3) and glycogen (Table 4) increased with months in storage under FTR but not STR (Fig. 3a–b). The top ranked model for trehalose was the null model, meaning trehalose levels were similar across treatments and through time (Fig. 3c, Supplementary material Table S4). Lipid reserves decreased with months in storage under both temperature regimes but decreased significantly faster under FTR (Table 5, Fig. 3d). Besides time and temperature regime, *week* was the only other predictor included in a best-fit model for sugars, with late-season (week 6) bees having higher sugar levels overall (Supplementary material Table S2, Table 3).

**Table 3.** Summary of the best-mixed beta regression model explaining proportion of simple carbohydrates (sugars) in newly emerged adult female *Megachile rotundata*. Random terms were field plot and microplate. STR is the reference temperature regime. Week 2 is the reference week.

Effect	Estimate	S.E.	Z value	*P* value
Month	0.035	0.049	0.71	0.4785
Temp	0.114	0.041	2.74	0.0061
Week	0.118	0.041	2.85	0.0044
Month × temp	0.176	0.076	2.30	0.0212

**Table 4.** Summary of the best fit, mixed beta regression model explaining proportion of complex carbohydrates (glycogen) in newly emerged adult female *Megachile rotundata*. Random terms were field plot and microplate. STR is the reference temperature regime. Week 2 is the reference week.

Effect	Estimate	S.E.	*Z* value	*P* value
Month	−0.198	0.118	−1.68	0.0934
Temp	0.281	0.094	2.98	0.0028
Month × Temp	0.480	0.161	2.99	0.0028

**Table 5.** Summary of the best fit, mixed beta regression model explaining proportion of total lipids in newly-emerged adult female *Megachile rotundata*. Random terms were field plot and microplate. STR is the reference temperature regime.

Effect	Estimate	S.E.	*Z* value	*P* value
Month	−0.062	0.037	−1.70	0.0890
Month^2^	0.082	0.037	2.23	0.0256
Temp	−0.063	0.031	−2.04	0.0415
Month × Temp	−0.026	0.057	−0.46	0.6438
Month^2^ × Temp	−0.170	0.053	−3.21	0.0013

### Weight

**Figure 4.**
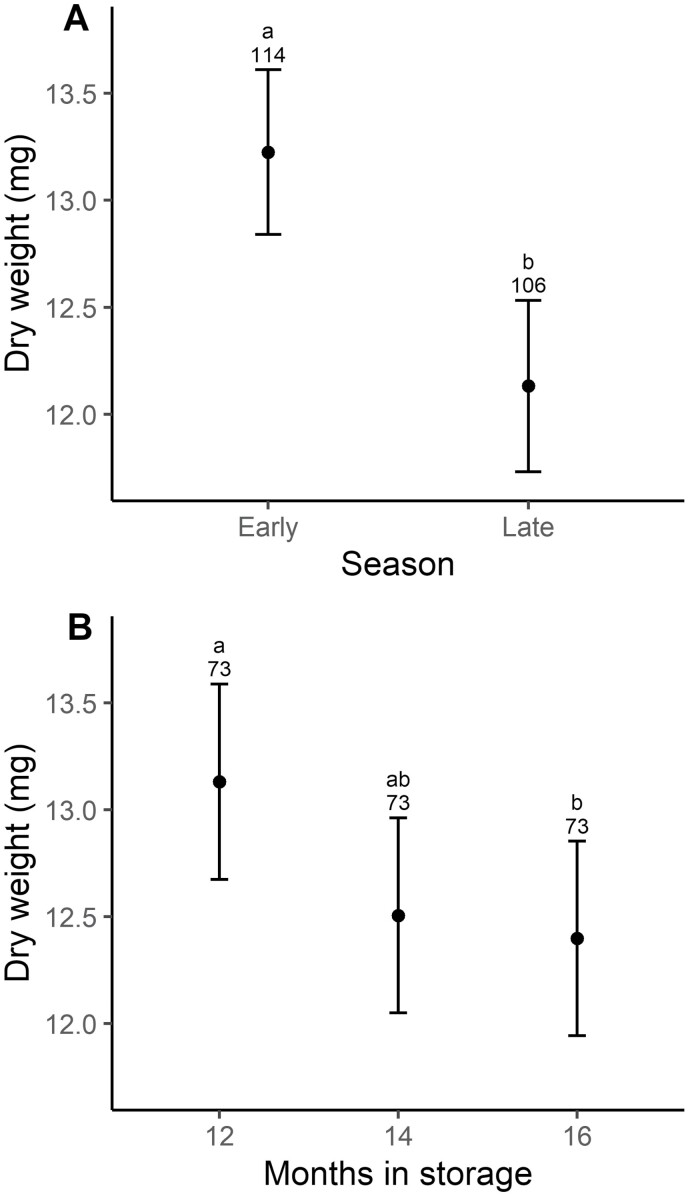
Marginal effects of (A) nest timing (early or late in the season) and (B) months in cold storage were tested on adult female *Megachile rotundata* mass. Data are estimated means ± 95% CI. The Numbers above the error bars represent sample sizes. Letters above error bars indicate a significant difference at *α* = 0.05.

On average, late-season female bees were smaller than early-season females (*t*_208.8 _= −3.164, *P* = 0.0018; Fig. 4a). Neither temperature regime (*t*_207.5 _= 1.588, *P* = 0.114) nor wildflower strips (*t*_8.7 _= 0.581, *P* = 0.576) influenced female mass. Emerged bees were significantly lighter in January 2019 than at baseline four months earlier, when temperature treatments were initiated, in September 2018 (*t*_208 _= 2.50, *P* = 0.0351; Fig 4b).

## Discussion

Our study combined field and lab experiments to test whether supplementing alfalfa fields with wildflower strips affected *M. rotundata* overwintering success under two extended cold storage temperature regimes. FTR dramatically improved *M. rotundata* overwintering survival, even when applied after a year of static cold. Although we observed no measurable effect of supplementing alfalfa fields with late-blooming wildflower strips on *M. rotundata* nutritional status or overwintering success, timing of nest establishment (which reflected the timing of cell-provisioning and the larval feeding period) emerged as a key factor. Our investigation combining the effects of the timing of cell provisioning, overwintering cold treatments and macronutrient stores extends our physiological understanding of the benefits of FTR.

A growing body of research demonstrates benefits of enhancing farmland with wildflowers for managed (Decourtye *et al.* 2010; Alaux *et al.* 2017) and wild (Morandin and Kremen 2013; Williams *et al.* 2015; Venturini *et al.* 2017) pollinators. Here, we diversified alfalfa fields with wildflower strips to test whether increased late-season forage opportunities would improve *M. rotundata* overwintering success and nutritional stores under extended cold storage. Contrary to predictions, adding wildflower strips had no effect on overwintering survival. Emergence rates were similar between plots with and without wildflower strips regardless of whether wildflowers strips were blooming. Additionally, after a year in cold storage, *M. rotundata* adult female carbohydrate and lipid stores did not differ in plots with or without wildflower strips. Although field and cage studies show that *M. rotundata* collects (O’Neill *et al.* 2004; O’Neill and O’Neill 2011), and even prefers (Stubbs *et al.* 1994; Horne 1995; Small *et al.* 1997), non-alfalfa pollen sources, visitation to the particular flowers in our wildflower strips was low. We, therefore, conclude that foraging *M. rotundata* did not use the wildflower strips to an extent that would affect nutritional stores or overwintering success of their offspring. Pollen provides the nutrition upon which offspring develop: the size and composition of the pollen provision affects offspring size and nutritional stores (i.e., lipid, protein, and carbohydrates) available for overwintering survival, development, and performance upon emergence (Roulston and Cane 2002; Chole *et al.* 2019; Parreño *et al.* 2022). Studies of honey bees and bumble bees show diverse pollen diets improved bee health, reproduction, and resilience to stress, by providing a complete set of essential nutrients (Vaudo *et al.* 2015) and increasing the chances of consuming pollen with pharmaceutical properties (Richardson *et al.* 2015). Unlike eusocial honey bees and bumble bees, whose offspring feed from a mix of pollen collected by generalist foragers, alfalfa leafcutting bee offspring feed on pollen collected by a single female. Constrained by a smaller foraging range, alfalfa leafcutting bees may not have the ability to take advantage of as great a diversity of pollens. Additionally, a choice experiment shows that pollen preference of *M. rotundata* does not necessarily lead to increased offspring survival (Horne 1995). Female *M. rotundata* at another site in Montana collected non-alfalfa pollen even when nest shelters sat amid alfalfa fields, and they included a greater percentage of non-alfalfa pollen later in the season (O’Neill *et al.*, 2004; O’Neill and O’Neill, 2011). However, in this study, late-season larvae did not produce adults of higher quality. We conclude that for wildflower strips to increase *M. rotundata* sustainability, further development of the floral composition of strips may be needed to cater specifically to *M. rotundata* success. To investigate whether *M. rotundata* overwintering success improves with diversification of diet per se, future study is needed in a more controlled setting (i.e., cage) that promotes diversification of pollen use and controls for other confounding variables, such as maternal influences.

Fluctuating temperature regimes have been shown to increase overwintering survival in insects across six orders (Colinet *et al.* 2018). The positive effect of FTR on *M. rotundata* winter survival under normal and extended cold storage has also been previously documented (Rinehart *et al.* 2013); however, the present study reveals for the first time that this increased overwintering success is associated with FTR-associated changes in macronutrient stores. Sugars and glycogen levels increased over time in females emerging from FTR, but that was not the case for those in STR. The leading hypothesis for benefits of FTR on overwintering success is that daily warm pulses afford recovery and repair from chill injury associated with a general loss of homeostasis during preceding cold periods (Colinet *et al.* 2018). Observed increases in stored and available carbohydrates could help to reestablish ion balance and mitigate damage from osmotic and ionic stress (Colinet *et al.* 2015). Aside from observable increases in offspring winter survival, higher energy reserves at emergence have foreseeable benefits for flight, successful mating, nest establishment, and ultimately, fitness. Increasing availability of energy stores began months before the emergence of success deviated between cold treatments. Elevated levels of carbohydrates in FTR-treated *M. rotundata* observed in this study complement previous findings by Torson *et al.* (2015) showing higher expression levels of genes involved in metabolism under FTR.

Most overwintering insects rely on stored lipids to survive an extended period of cold and to fuel post-winter activities and egg production (Sinclair and Marshall 2018). Increased metabolic activity under FTR should, theoretically, deplete stored energy reserves faster than static cold treatments. Previous work, however, comparing depletion of lipid reserves under STR and FTR reveals that some insects use more lipids in FTR while others do not (Pullin and Bale 1989; Ismail et al. 2010, 2013). In this study *M. rotundata* lost more lipid reserves under FTR than under STR, so FTR poses a potential energetic cost for this pollinator. The amount of lipid stores remaining after overwintering has been shown to influence post-winter fitness, by influencing reproductive success and flight performance (reviewed in Sinclair and Marshall, 2018). Indeed lipid stores of *M. rotundata* females decline rapidly during the first week after emergence, coinciding with increases in the volume of oocytes in their ovaries (O’Neill *et al.* 2015). Because *M. rotundata* lipid reserves average about 30% body mass versus 8% body mass of honey bees (Buckner *et al.* 2004), the cost of recovery and/or repair of FTR may be relatively low. When comparing *M. rotundata* stored for a single winter under STR with *M. rotundata* stored for an additional 12 months (extended storage) under FTR, flight ability and *respiratory capacity were similar for newly emerged adults (*Bennett *et al.* 2013). Whether FTR’s increased energetic cost reduces other fitness measures (e.g., reproduction and life span) is unknown and warrants further study. Our macronutrient results support a growing body of research showing that bees exposed to fluctuating temperatures are physiologically distinct from those maintained under constant cold storage (Bennett *et al.* 2013; Yocum *et al.* 2018; Cambron *et al.* 2021). To better understand the mechanistic benefits of FTR for overwintering insects, further energetic studies that measure both macronutrient stores and metabolism are needed.

High pre-emergent adult mortality observed in STR but not FTR (Fig. 2c) supports the hypothesis that FTR mitigates neuromuscular cold injury. By the end of temperature treatments, 21% and 5% of bees that failed to emerge had fully developed into adults under STR and FTR, respectively. Failed ion homeostasis can lead to neuromuscular changes, chill injury, and death (Overgaard and MacMillan 2017). Flesh flies fail to emerge as a result of neuromuscular injury from critically low temperatures (Yocum *et al.* 1994). For the hemipteran, *Pyrrhocoris apterus,* and the beetle *Alphitobius diaperinus*, FTR’s warm spells allowed ion gradients to re-establish across membranes (Koštál *et al.* 2007). Bennett *et al.* (2013) found *M. rotundata* overwintered in extended FTR to have similar respiratory capacity and flight ability as those stored for a normal overwintering cycle under STR. Under FTR, M. *rotundata* also expressed an increased abundance of transcripts involved in counteracting disruption of ion homeostasis (Torson *et al.* 2015). Our findings, taken with those of previous research on *M. rotundata,* provide evidence for the important role FTR plays in maintaining neuromuscular function under extended cold storage.

Contrary to prediction, *M. rotundata* offspring produced early in the season had higher survival (Fig. 2b) and body mass (Fig. 4a) than those produced late in the season. Early diapausing *Osmia lignaria* (a similarly solitary, stem-nesting bee in the family Megachilidae) had lower overwintering survival associated with depleted energy reserves and greater exposure to temperature extremes compared to their late diapausing kin (Sgolastra *et al.* 2011). Similar costs of lengthened prewinter and diapause periods have been reported for apple maggot (Feder *et al.* 1997) and a parasitoid wasp (Ellers and Van Alphen 2002). However, other insects, such as the Colorado potato beetle, have also demonstrated no cost of increased periods of dormancy (Peferoen *et al.* 1981; Jansson *et al.* 1989). *Megachile rotundata’s* relatively high prepupal lipid stores (Buckner *et al.* 2004) may ensure adequate reserves during lengthened dormancy. While *M. rotundata* overwintering survival did not increase with added wildflower strips, seasonally-influenced survival differences may still be mediated by floral resources, specifically those presented by the alfalfa crop. Overwintering survival increases with body size (Hahn and Denlinger 2007), which is influenced by the quality and quantity of the pollen provision (Roulston and Cane 2002). Bees produced early in the season would have foraged primarily on a monocrop of alfalfa flowers, with alfalfa floral resources peaking in the early weeks after release and decreasing with each week following (Delphia et al. *in prep*). Alfalfa pollen is relatively rich in protein and amino acids, which may facilitate *M. rotundata*’s healthy reliance on a diet comprised mostly of alfalfa pollen (Taha *et al.* 2019). Fewer alfalfa flowers may have resulted in smaller provisions and consequently smaller body mass and survival of late season bees (Peterson and Roitberg 2006). *Megachile rotundata* females accumulate considerable wing wear as the flight season progresses (O’Neill *et al.* 2015), which may decrease their foraging efficiency in late summer, further reducing provision mass size. Alternatively, decreasing offspring quality through time may be maternally influenced. As maternal age increases, offspring performance decreases in several insect species (Mohaghegh *et al.* 1998; Kern *et al.* 2001; Fox *et al.* 2003). Whether floral resources or maternal effects are driving overwintering survival or not, our results support earlier work showing early and late season bees are physiologically distinct as demonstrated by differential expression of various diapause regulating genes that impact metabolism such as Forkhead box protein O, Samui, and the proto-oncogene Myc (Yocum *et al.* 2018; Cambron-Kopco *et al.* 2022).

## Conclusion

We aimed to increase forage diversity by providing wildflower strips, but the use of wildflower strips by *M. rotundata* was low and did not increase *M. rotundata* macronutrient stores or overwintering survival. The low sustainability of managed *M. rotundata* populations in the United States may be driven by factors other than forage diversity, such as availability of nesting materials, storage handling and *conditions, parasites, and high stocking densities (*Pitts-Singer and Cane 2010). *Megachile rotundata* offspring fed on provisions early in the season were larger and had higher survival rates than their late-season counterparts. Healthy reliance on a single forage may be a result of efficient foraging on a high density of alfalfa flowers that also provide high-quality protein (Taha *et al.* 2019). Differences between FTR and STR survival and macronutrient content under extended cold storage open inquiry into a possible energetics mechanism behind the dramatic emergence success of *M. rotundata* under FTR. Exposure to warm pulses during extended cold storage increased accessible and stored carbohydrates while decreasing lipid stores, revealing a potential energy cost of FTR in our study organism. A closer look at specific lipids, reproduction, and post-emergence performance would help us better understand potential tradeoffs of FTR. Our findings build upon previous work investigating distinctions between STR- and FTR- treated bees to better understand mechanisms leading to FTR’s benefits for overwintering survival for *M. rotundata* and other insects.

## Supplementary Material

nvac062_suppl_Supplemental_Materials

## Data Availability

Data from this study are available from the Dryad Digital Repository: https://doi.org/10.5061/dryad.8pk0p2nqw (Park, 2022).
