# Supplementary material for: Effects of Temperature and Wildflower Strips on Survival and Macronutrient Stores of the Alfalfa Leafcutting Bee (Hymenoptera: Megachilidae) Under Extended Cold Storage
Source: Environ Entomol. 2022 Aug 14;51(5):958–68. doi: 10.1093/ee/nvac062 (PMC9585370; doi:10.1093/ee/nvac062)
Supplement: nvac062_suppl_Supplemental_Materials [file nvac062_suppl_supplemental_materials.docx]

Supplemental Information

Table S1. Mixed logistic regression models testing effects of months in cold storage, temperature regime (STR or FTR), wildflower strips (P/A) and nesting week on *Megachile rotundata* offspring emergence. Model rank, variables, number of estimable parameters (K), log-likelihood (log [L]), Akaike’s Information Criterion (AICc), ΔAIC, and Akaike weights (ω_i_) for top 5 logistic regression models. Models were ranked by AIC score from 13 candidate models.

| Rank | Model variables | K | Log (L) | AICc | ΔAIC | ωi |
| --- | --- | --- | --- | --- | --- | --- |
| 1 | month, month^2^, month^3^, temp, week, month × week, month × temp, month^2^ × temp, month^3^ × temp | 11 | -1819.35 | 3660.7 | 0.00 | 0.688 |
| 2 | month, month^2^, month^3^, temp, week, month × temp, month^2^ × temp, month^3^ × temp | 10 | -1822.00 | 3664.0 | 3.30 | 0.132 |
| 3 | month, month^2^, month^3^, temp, week, wildflower, month × week, wildflower × week, month × temp, month^2^ × temp, month^3^ × temp | 13 | -1819.26 | 3664.6 | 3.84 | 0.101 |
| 4 | month, month^2^, month^3^, temp, week, wildflower, month × temp, month^2^ × temp, month^3^ × temp | 11 | -1821.92 | 3665.9 | 5.14 | 0.053 |
| 5 | month, month^2^, month^3^, temp, week, wildflower, wildflower × week, month × temp, month^2^ × temp, month^3^ × temp | 12 | -1821.92 | 3667.9 | 7.14 | 0.019 |

Table S2. Mixed beta regression models testing effects of months in storage, temperature regime, wildflower strips and nesting week on proportion sugars of female, adult *Megachile rotundata*. Model rank, variables, number of estimable parameters (K), log-likelihood (log [L]), Akaike’s Information Criterion (AICc), ΔAIC, and Akaike weights (ω_i_) for top 5 beta regression models. Models were ranked by AIC score from 16 candidate models.

| Rank | Model variables | K | Log (L) | AICc | ΔAIC | ωi |
| --- | --- | --- | --- | --- | --- | --- |
| 1 | month, temp, week, month × temp | 33 | 843.61 | -1609.2 | 0.00 | 0.626 |
| 2 | month, temp, week, wildflower, month × temp | 34 | 843.65 | -1606.4 | 2.72 | 0.161 |
| 3 | month, temp, month × temp | 32 | 839.77 | -1604.3 | 4.90 | 0.054 |
| 4 | month, temp, week, month × temp, month × week | 35 | 843.80 | -1603.9 | 5.26 | 0.039 |
| 5 | month, temp, wildflower, week, month × temp, wildflower × week | 35 | 843.66 | -1603.6 | 5.55 | 0.031 |

Table S3. Mixed beta regression models testing effects of months in storage, temperature regime, wildflower strips and nesting week on proportion glycogen of female, adult *Megachile rotundata*. Model rank, variables, number of estimable parameters (K), log-likelihood (log [L]), Akaike’s Information Criterion (AICc), ΔAIC, and Akaike weights (ω_i_) for top 5 beta regression models. Models were ranked by AIC score from 16 candidate models.

| Rank | Model variables | K | Log (L) | AICc | ΔAIC | ωi |
| --- | --- | --- | --- | --- | --- | --- |
| 1 | month, temp, month × temp | 14 | 1015.16 | -2000.2 | 0.00 | 0.439 |
| 2 | temp | 10 | 1009.58 | -1990.1 | 2.11 | 0.153 |
| 3 | month, temp, month × temp, week | 15 | 1015.23 | -1998.0 | 2.18 | 0.148 |
| 4 | month, temp, month × temp, wildflower | 15 | 1015.22 | -1998.0 | 2.20 | 0.146 |
| 5 | month, temp, month × temp, wildflower, week | 16 | 1015.30 | -1995.8 | 4.40 | 0.049 |

Table S4. Mixed beta regression models testing effects of months in storage, temperature regime, wildflower strips and nesting week on proportion trehalose of female, adult *Megachile rotundata*. Model rank, variables, number of estimable parameters (K), log-likelihood (log [L]), Akaike’s Information Criterion (AICc), ΔAIC, and Akaike weights (ω_i_) for top 5 beta regression models. Models were ranked by AIC score from 16 candidate models.

| Rank | Model variables | K | Log (L) | AICc | ΔAIC | ωi |
| --- | --- | --- | --- | --- | --- | --- |
| 1 | null | 27 | 2307.46 | -4553.0 | 0.00 | 0.430 |
| 2 | temp | 28 | 2307.70 | -4550.8 | 2.15 | 0.147 |
| 3 | month, temp, month × temp | 32 | 2312.86 | -4550.4 | 2.63 | 0.115 |
| 4 | month, temp, week, month × temp, month × week | 35 | 2316.85 | -4549.9 | 3.07 | 0.093 |
| 5 | month, temp, week, month × temp | 33 | 2313.94 | -4549.8 | 3.24 | 0.085 |

Table S5. Mixed beta regression models testing effects of months in storage, temperature regime, wildflower strips and nesting week on proportion total lipids of female, adult *Megachile rotundata*. Model rank, variables, number of estimable parameters (K), log-likelihood (log [L]), Akaike’s Information Criterion (AICc), ΔAIC, and Akaike weights (ω_i_) for top 5 beta regression models. Models were ranked by AIC score from 16 candidate models.

| Rank | Model variables | K | Log (L) | AICc | ΔAIC | ωi |
| --- | --- | --- | --- | --- | --- | --- |
| 1 | month, month^2^ , temp, month × temp, month^2^ × temp | 32 | 434.24 | -793.2 | 0.00 | 0.403 |
| 2 | month, temp, wildflower | 33 | 434.47 | -790.9 | 2.31 | 0.127 |
| 3 | month, temp, week | 33 | 434.40 | -790.7 | 2.45 | 0.118 |
| 4 | null | 27 | 428.28 | -789.4 | 2.85 | 0.097 |
| 5 | month | 29 | 436.37 | -789.0 | 3.79 | 0.061 |
